# Knowledge and attitudes of nurses towards telenursing in cardiac care in Al-Ahsa, Saudi Arabia: a cross-sectional study

**DOI:** 10.3389/fcvm.2025.1506441

**Published:** 2025-03-12

**Authors:** Dalal Abdulrahman Alsultan, Sahbanathul Missiriya Jalal

**Affiliations:** Department of Nursing, College of Applied Medical Sciences, King Faisal University, Al-Ahsa, Saudi Arabia

**Keywords:** telenursing, knowledge, attitudes, nurses, heart failure, telehealth

## Abstract

**Objectives:**

Telenursing in cardiac care leverages technology to support self-care and optimize outcomes for heart failure patients during and beyond the pandemic. This study aims to explore nurse's knowledge and attitudes towards telenursing in cardiac care, examine the correlation between knowledge and attitude, and associate knowledge levels with selected demographic variables.

**Methods:**

A cross-sectional study was conducted, and 149 nurses from Prince Sultan Cardiac Care Center hospitals were randomly selected. The data were collected through a structured questionnaire, including socio-demographic characteristics, knowledge, and attitudes about telenursing.

**Results:**

The mean age of the nurses was 36.17 ± 6.5 years. Most nurses (64.4%) held a bachelor's degree, with 71.8% working as staff nurses. Nearly half (47.6%) had 6–9 years of professional experience. Among them, 15.4% had good knowledge, 63.8% had average knowledge, and 20.8% had poor knowledge, with a total mean knowledge score of 14.92 ± 3.2. Overall, nurses had positive attitudes towards telenursing (*p* < 0.05). Knowledge scores were significantly associated with age, education, and professional experience (*p* < 0.05).

**Conclusion:**

Strengthening nurse's understanding of telenursing is vital. Focused educational and training programs are imperative to enhance knowledge about telenursing in cardiac care and alleviate the healthcare system's economic burden.

## 1 Introduction

Telehealth refers to the use of telecommunications tools, such as video conferencing, mobile health apps, and remote monitoring devices, to provide healthcare services remotely. It makes it easier for patients and healthcare professionals to communicate and ensure continuity of care despite geographic distance. This approach offers several benefits, including increased access to healthcare, reducing hospital service costs, and improving patient health outcomes (1). The world health organization (WHO) recognizes the significance of telehealth and advocates for its adoption, particularly in regions with limited resources or healthcare access. It helps countries to assess its impact on healthcare accessibility and delivery (2).

Telenursing, a vital component of telehealth, leverages information and communication technologies to remotely meet the healthcare needs of the patients (3). Through this innovative approach, nurses can digitally provide professional care services. In remote nursing, nurses use a range of technologies such as the internet, computers, phones, digital tools, and remote monitoring devices to provide essential nursing care components, such as assessment, planning, intervention, and outcome evaluation. This model allows nurses to communicate with patients in real-time, offer health education, address queries, and provide advice (4).

Telenursing technologies enable nurses’ to efficiently communicate and monitor through phone calls or text messages, thereby facilitating real-time interactions and immediate responses. Remote monitoring devices track vital signs and other physical measurements, ensuring the continuous observation of patient health (5). Telenursing and telemedicine are useful in cardiac care, especially for patients with heart failure (HF). According to a previous meta-analysis, telenursing interventions can improve the quality of life among patients with HF, thereby indicating that remote monitoring and education should be included as part of standard nursing procedures (6). Telemonitoring techniques, particularly those that integrate pharmaceutical support with mobile health technologies, are associated with lower rates of all-cause mortality and hospitalization among patients with chronic HF. These results highlight how telehealth interventions may lower healthcare utilization and improve patient outcomes (7). These advancements have resulted in improved healthcare accessibility while reducing costs, making telenursing particularly beneficial for managing chronic conditions such as HF (8, 9).

According to the American Heart Association (AHA), HF is a chronic condition in which the heart cannot pump enough blood to support body functions (10). HF is caused by irregularities in the structure and function of the heart, which results in poor ventricular filling or blood ejection, and can lead to hemodynamic imbalance. Owing to these structural or functional abnormalities, HF leads to symptoms such as dyspnea, fatigue, fluid retention, exercise intolerance, peripheral edema, and pulmonary congestion (11). Managing HF is a major challenge due to its long-term presence and complex treatment requirements. Continuous monitoring is necessary to evaluate disease progression, treatment response, and early signs of decompensation. This poses a significant challenge, as continuous monitoring of symptoms, vital signs and biomarkers are essential for guiding treatment modifications and preventing worsening of symptoms (12). Healthcare systems face difficulties in preventing complications and managing patients with HF (13). Consequently, it is essential to investigate alternative methods to enhance patient outcomes and self-care practices.

Virtual cardiac clinics have emerged as an innovative option for delivering health education and remote surveillance for patients with HF (14). Telenursing is a highly innovative approach to healthcare delivery aimed at improving the quality of life of patients with cardiac issues, reducing readmission rates, and ultimately lowering mortality rates (15). Through this method, nurses can easily observe and follow up on patients with HF and provide health education without being hindered by geographical constraints. Another important aspect is educating and reassuring patients as managing HF requires lifestyle changes, adherence to medication, and practicing self-care. Providing patients with information about their condition, such as dietary limitations, fluid control, and identification of worsening symptoms, is crucial for enabling them to play an active role in their treatment (16).

However, obstacles remain in the widespread use of telenursing for cardiac care. A comprehensive review found that these obstacles include issues such as data security, lack of established standards, and technological restrictions. These issues must be resolved for the successful and sustainable implementation of telenursing services in cardiac care (17). Moreover, adherence to treatment regimens for HF can be challenging because of the need for complex medication schedules and lifestyle modifications. Failure to follow medical recommendations can result in ineffective symptom management, frequent hospital stays, and adverse outcomes. Nonetheless, further research is needed to explore how virtual HF clinics can be utilized as a new nursing intervention to provide patients with individualized education and supervision (18).

Understanding the perspectives of nurses is crucial, as it influences their readiness to adopt and use virtual nursing clinics. Recognizing obstacles or difficulties that nurses may encounter can help stakeholders customize interventions and tactics to tackle these issues and encourage the effective introduction of virtual cardiac clinics. Moreover, the results may help shape interventions and educational initiatives aimed at enhancing the utilization of virtual clinics in similar healthcare settings (6). Therefore, the researchers aimed to assess knowledge and attitudes toward telenursing in cardiac care, correlating knowledge with attitudes and examining the relationship between knowledge levels and selected demographic variables at the cardiac care center.

## 2 Materials and methods

### 2.1 Study design and participants

A cross-sectional research design was used to gather data on knowledge and attitudes towards telenursing in cardiac care among nurses working at Prince Sultan Cardiac Care Center Hospitals (PSCCCH) in Al-Ahsa, Kingdom of Saudi Arabia. The nurses were initially identified based on inclusion criteria to determine their eligibility for participation. The inclusion criteria encompassed nurses working at PSCCCH, including both genders, with various nationalities, obtained minimum diploma in nursing educational qualification, and one year or more clinical nurse experiences. The nurses with less than one year of experience, those not interested to participate in this research, those who filled out the questionnaire incompletely, and those who withdrew during the study were excluded.

The sample size was calculated using Slovin's formula, based on outcomes from a previous study (5). Assuming 50% of the study population had awareness of telenursing, with a 5% margin of error and a 95% confidence level, and considering a finite population of 335 nurses, the minimum required sample size was determined to be 182 participants. This ensured the study had adequate power to detect meaningful differences and generate reliable conclusions.

After verifying the eligibility from the professional databases at PSCCCH, 292 eligible nurses were identified. Using simple randomization sampling with the assistance of computer-generated software, 190 nurses were approached through email with institutional collaboration. Despite these efforts, not all eligible nurses participated in the study due to personal reasons or workload constraints. However, 149 nurses expressed their interest in participating in the study and completed the data collection.

Ethical clearance was obtained from the Research Ethics Committee of the Deanship of Scientific Research, King Faisal University, Saudi Arabia (KFU-REC-2023-JUN-ETHICS953), and the study adhered to the guidelines of the Declaration of Helsinki. Ethical approval was also secured from the institutional review board of PSCCCH. Written informed consent was obtained from all participating nurses before data collection, and assurance was ensured to protect their anonymity and confidentiality, confirming no risk and voluntary participation. The data was collected through a structured questionnaire, which included sections on socio-demographic characteristics, knowledge, and attitudes of nurses regarding telenursing.

### 2.2 Questionnaire development

The data collection tool was prepared by the researcher after reviewing relevant national and international literature to meet the study's objectives. The structured self-administered tool was validated by seven panels of experts from the medical and nursing fields. To ensure reliability and validity, the questionnaire underwent a pilot test on 10% of the total sample to evaluate its clarity, feasibility, and applicability, as well as the time needed to complete the tool. The reliability of the tool was tested by measuring their internal consistency using Cronbach's alpha coefficient (*r* = 0.934) through the pilot project. Since the survey tool was developed in English and administered to participants proficient in the language, no linguistic issues arose during the study. This ensured clarity in communication and accurate responses, eliminating any potential language barriers.

The self-administered questionnaire consisted of three parts. The first part collected socio-demographic data, including age, gender, highest educational qualification, designation, years of experience, nationality, and participation in training programs on telenursing. Training in telenursing was provided to all participants as part of their work. The second part assessed knowledge about telenursing (19) using 24 multiple-choice questions. Each question had four options, with one correct answer scored as one and an incorrect answer scored as zero. Scores were categorized as follows: 75%–100% (18–24) for good knowledge, 50%–74% (12–17) for average knowledge, and below 50% (less than 12) for poor knowledge. The third part evaluated the attitude of nurses towards telenursing (8) using a Likert scale with eight items ranging from 1 (strongly disagree) to 5 (strongly agree). For negatively phrased statements, scores were reversed during data entry. The overall attitude score was calculated by summing the individual scores, and the mean was computed by dividing the total score by the number of participants (149). Attitude scores below the mean were classified as negative, while scores at or above the mean were classified as positive.

### 2.3 Data collection

Data was collected digitally using Google Forms, with reminders sent via email to non-respondents. The survey was accessible for four weeks, allowing nurses to respond at their convenience in a single attempt. The time required to complete the questionnaire ranged from 25 to 30 min. The study objectives were explained to the nurses before data collection commenced. Data collection occurred between September 2023 and March 2024. To maintain privacy and confidentiality, the collected data were electronically encrypted and stored securely, with access restricted to the principal investigator through a password.

### 2.4 Statistical analyses

The data were analyzed using IBM SPSS Statistics for Windows, Version 21.0 (IBM Corp., Armonk, NY, USA). Descriptive statistics were used to calculate frequencies, percentages, means, and standard deviations. A chi-square test was conducted to assess the associations between the nurse's knowledge regarding telenursing and their demographic characteristics, with a significance level of *p* ≤ 0.05. Additionally, the correlation coefficient (*r*) test was used to measure the relationship between the level of knowledge and attitudes of nurses regarding telenursing (*p* ≤ 0.05).

## 3 Results

### 3.1 Socio-demographic characteristics

In this survey, a total of 149 nurses participated, as shown in Table 1. Among them, 91.9% were female. Half of the participants (51.7%) were aged between 30 and 39 years, with a mean age of 36.17 ± 6.5 years. Regarding the highest level of qualification, 96 participants (64.4%) held a bachelor's degree. In terms of designation, 71.8% of the participants were staff nurses, 18.8% were nurses in charge, and 9.4% were head nurses. Most nurses, 71 (47.6%) had 6–9 years of professional experience. Indian nurses represented the largest group, accounting for 94 participants (63.1%), remaining nurses were from other nationalities, including Saudi nurses. Only a small proportion, 25 nurses (16.8%), had participated in training on telenursing, while the majority, 124 nurses (83.2%), had not attended any training sessions related to telenursing.

**Table 1:** Socio-demographic characteristics of the nurses (*n* = 149).

Variables		Number	Percentage (%)
Age (years)	20–29 years	29	19.4
	30–39 years	77	51.7
	40–49 years	38	25.5
	50–59 years	5	3.4
Gender	Male	12	8.1
	Female	137	91.9
Educational qualification (highest)	Diploma	29	19.5
	Bachelor	96	64.4
	Master	24	16.1
Designation	Nurse	107	71.8
	Nurse in charge	28	18.8
	Head nurse	14	9.4
Professional experience	1–3 years	41	27.5
	4–6 years	71	47.6
	6–9 years	32	21.5
	10 years and above	5	3.4
Nationality	Saudi	7	4.7
	Egyptian	1	0.7
	Sudanese	1	0.7
	Pilipino	41	27.4
	Indian	94	63.1
	Jordanian	1	0.7
	Pakistani	4	2.7
Training participation on telenursing	Yes	25	16.8
	No	124	83.2

### 3.2 Knowledge level of nurses regarding telenursing

**Figure 1:**
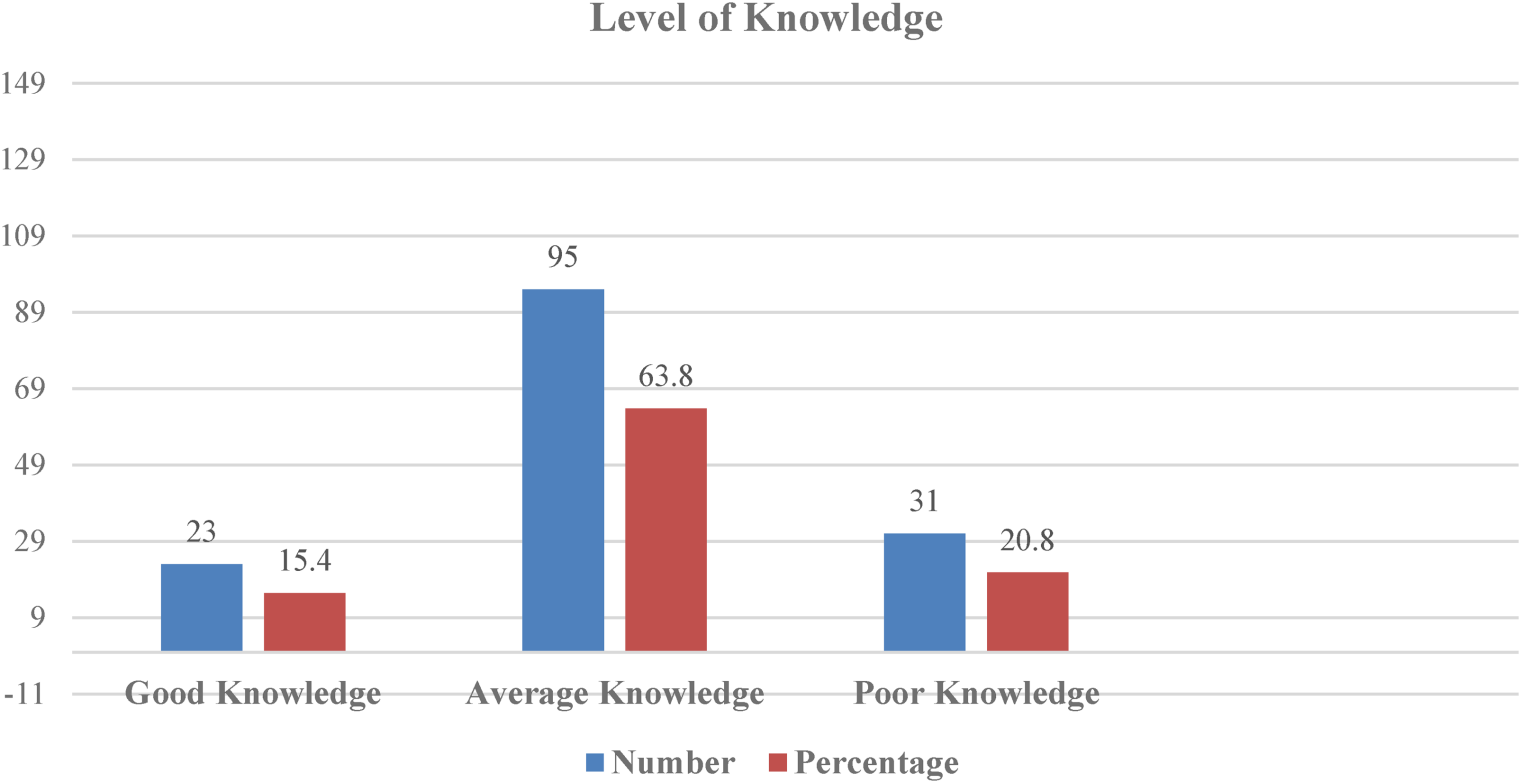
Frequency distribution of knowledge level of nurses regarding telenursing.

The frequency distribution of the knowledge level of nurses regarding telenursing is depicted in Figure 1. Among the 149 nurses surveyed, 23 (15.4%) demonstrated good knowledge, 95 (63.8%) had average knowledge, and 31 (20.8%) had poor knowledge. The mean scores for knowledge about telenursing across various subsections are presented in Table 2. The total mean knowledge score was 14.92 ± 3.2. The highest mean score was observed in the subsection on general information about telenursing (2.81 ± 0.96), while the lowest mean score was recorded in the subsection on risk management in telenursing (1.56 ± 1.16).

**Table 2:** Mean scores of nurse's knowledge about telenursing as per subsections. (*n* = 149).

Knowledge questionnaire subsections	Number of items	Range	Mean ± SD
General information about telenursing	4	0–4	2.81 ± 0.96
Identification of clients need in telenursing	3	0–3	1.97 ± 1.08
Technology choice and usage in telenursing	4	0–4	2.35 ± 1.58
Risk management in telenursing	3	0–3	1.56 ± 1.16
Privacy, confidentiality and security of client's documents	3	0–3	1.78 ± 1.08
Therapeutic communication	3	0–3	1.98 ± 1.21
Documentation and referrals	4	0–4	2.47 ± 1.37
Total	24	0–24	14.92 ± 3.2

SD, standard deviation.

### 3.3 Attitude of nurses regarding telenursing

The frequency distribution of nurses' attitudes toward telenursing is presented in Table 3. Among the participants, 65 (43.6%) strongly agreed that telenursing provides comprehensive healthcare services, and 57 (38.3%) believed that it enhances communication among providers. Regarding whether telenursing increases timely access to information, 53 (35.6%) held a neutral stance. Concerning the improvement of tracking patient health status, 40 participants (26.8%) agreed with this statement. Nearly half of the participants, 67 (45%), strongly agreed that telenursing poses a threat to patient privacy, while 36 (24.2%) strongly agreed that it increases medical costs. Additionally, 43 participants (28.9%) believed that telenursing improves the quality of healthcare information, and 48 (32.2%) disagreed that telenursing reduces medical errors.

**Table 3:** Frequency distribution of attitude level of nurses regarding telenursing (*n* = 149).

Attitude	Strongly agree	Agree	Neutral	Disagree	Strongly disagree
	*N* (%)	*N* (%)	*N* (%)	*N* (%)	*N* (%)
Provide comprehensive healthcare services	65 (43.6)	44 (29.5)	13 (8.7)	16 (10.7)	11 (7.4)
Increase communication among providers	57 (38.3)	35 (23.5)	29 (19.5)	21 (14.1)	7 (4.7)
Increase timely access to information	56 (37.6)	20 (13.4)	53 (35.6)	11 (7.4)	9 (6)
Improve tracking of patient health status	56 (37.6)	40 (26.8)	28 (18.8)	10 (6.7)	15 (10.1)
Threatening patient privacy^a^	67 (45)	36 (24.2)	25 (16.8)	14 (9.4)	7 (4.7)
Increase medical costs^a^	36 (24.2)	21 (14.1)	73 (49)	13 (8.7)	6 (4)
Improve quality of healthcare information	27 (18.1)	43 (28.9)	35 (23.5)	28 (18.8)	16 (10.7)
Reduce medical errors	11 (7.4)	23 (15.4)	28 (18.8)	48 (32.2)	39 (26.2)

^a^ Negatively phrased statements.

A significant positive correlation (*r* = 0.191) was observed between nurses’ knowledge and attitudes toward telenursing, as shown in Table 4 (*p* < 0.05). It highlights the importance of educational interventions to improve attitudes by enhancing knowledge. This finding provided a basis for future studies to explore additional factors that may strengthen the relationship between knowledge and attitudes toward telenursing.

**Table 4:** Correlation between knowledge and attitude of nurses regarding telenursing (*n* = 149).

Items	R value	*p* value
Knowledge	0.191*	0.020
Attitude		

* Significant at (*p* < 0.05).

### 3.4 Association between levels of knowledge about telenursing with the demographic variables

Table 5 presents the association between knowledge levels and demographic variables. The knowledge score was found to be significantly associated with age, educational qualification, and professional experience (*p* < 0.05).

**Table 5:** Association of level of knowledge with the demographic variables (*n* = 149).

Variables		Good	Average	Poor	*X* ^2^
Age (years)	20–29 years	1	14	14	*X*^2^ = 20.763 *p* = 0.002*
	30–39 years	13	53	11	
	40–49 years	9	24	5	
	50–59 years	2	2	1	
Gender	Male	1	10	1	*X*^2^ = 2.187 *p* = 0.335 NS
	Female	22	85	30	
Educational qualification (highest)	Diploma	1	16	12	*X*^2^ = 13.0423 *p* = 0.011*
	Bachelor	18	64	14	
	Master	5	16	3	
Designation	Nurse	15	67	25	*X*^2^ = 4.3609 *p* = 0.3594 NS
	Nurse in charge	8	16	4	
	Head Nurse	2	10	2	
Professional experience	1–3 years	1	24	16	*X*^2^ = 16.1806 *p* = 0 0.013*
	4–6 years	16	46	9	
	6–9 years	6	21	5	
	10 years and above	1	3	1	
Training participation on telenursing	Yes	3	14	8	*X*^2^ = 2.3229 *p* = 0.313 NS
	No	20	81	23	

* Significant at (*p* < 0.05); NS, non-significant.

## 4 Discussion

This cross-sectional study was aimed to assess the knowledge and attitudes of nurses towards telenursing in cardiac care; only 15.4% demonstrated good knowledge. Nurses had mixed views on telemedicine and telenursing, recognizing benefits such as enhanced communication, while also expressing concerns about privacy and costs. In recent years, the healthcare industry has experienced significant transformation, particularly with the emergence of virtual nursing clinics. These innovative platforms have revolutionized patient care, especially in the management of chronic conditions such as cardiac diseases. The COVID-19 pandemic has highlighted challenges in accessing traditional medical and nursing care, emphasizing the need for alternative care delivery models. Nurse-led telehealth sessions have effectively addressed these gaps by improving access to essential nursing services and ensuring the continuity of care (20–22).

HF is a major public health concern, affecting approximately 5.7 million adults in Western countries, such as the United States, with a projected increase of 46% by the year 2030 (23). Approximately 3.6 million individuals are diagnosed with HF annually in Europe (24). The prevalence of HF is high in Asian countries (25). For example, in Indonesia, HF cases increased by 1.5% in 2018, equating to 15 cases per 1,000 individuals (26). Virtual nursing clinics offer a convenient and efficient solution for the follow up care of cardiac patients, enabling timely and personalized support from the comfort of their homes. Nurses play a pivotal role in these clinics, ensuring the continuity of care and fostering positive health outcomes in cardiac patients (16).

This study aimed to assess the knowledge and attitudes of cardiac care nurses towards telenursing. The findings revealed that most participants were female, with half of them aged between 30 and 39 years. Similar findings were noted regarding the predominance of female nurses in a study involving nursing students in Jordan that examined their perspectives on telenursing (27). Another study showed that nurses’ attitudes towards telemedicine and telenursing in cardiac care depend on training, age, and technological experience. In such studies, female nurses were often more likely to engage in new healthcare technologies such as telenursing, especially if they had access to sufficient training and resources (28). A comparable study on the perceived knowledge, self-confidence, and attitudes of nurses using telemedicine found that most respondents were aged 26–30 years (29). Age distribution is significant because it may influence nurses’ perceptions and adoption of telenursing technology. For nurses aged 30–39 years, often with more experience, may have different attitudes toward technological innovations than younger nurses, who may be more accustomed to integrating technology into their professional and personal lives. Recognizing these age-related differences is essential for interpreting the results and addressing the broader implications of telenursing adoption in diverse nursing populations.

In the current study, among 149 nurses, 15.4% demonstrated good knowledge, 63.8% had average knowledge levels, and 20.8% had poor knowledge. These findings align with a descriptive-quantitative cross-sectional study conducted among nurses working in the outpatient department of a selected private hospital in western Indonesia. That study found that most nurses perceived they had a moderate level of knowledge about telemedicine operations, some reported average self-confidence levels, and half expressed a neutral attitude toward telemedicine (30). Similarly, a multi-setting pre-test and post-test study conducted in the Netherlands revealed that an educational intervention significantly improved nurses’ knowledge, self-efficacy, and utilization of telehealth during training sessions (31). Telemedicine and telenursing can enhance the standard of care while reducing hospital burdens, patient suffering, ambulance calls, hospitalizations, and medical expenses. However, the implementation of these services is often hindered by a lack of knowledge, presenting significant challenges (32).

In the present study, an association was found between the level of knowledge and demographic variables, such as age, educational qualifications, and professional experience (*p* < 0.05). Similarly, an institution-based cross-sectional study conducted among health professionals working at referral hospitals identified factors associated with telemedicine knowledge. In that study, half of the professionals demonstrated good knowledge and high awareness of telemedicine services. Key factors such as the information-sharing culture, IT support staff, internet access for information, telemedicine training, and computer accessibility within the hospitals were significantly associated with the professionals’ awareness of telemedicine services (33). Another study aimed to determine the rate of telemedicine use and assess the awareness, knowledge, attitude, and skills of primary healthcare providers. The study found that the rate of telemedicine use was significantly associated with the levels of awareness (*p* < 0.05) (34).

Our current study revealed a strong positive association between nurses’ attitudes and knowledge levels toward telenursing. Despite this weak correlation, its practical significance shows that nurses who have a better understanding of telenursing might be more receptive to its implementation. The findings suggest that educational interventions such as workshops or online training could enhance both knowledge and attitudes, potentially improving the implementation of telenursing. A more extensive multi-site study that included the community also found a strong correlation between attitudes and knowledge. Approximately 50% of the participants believed that telehealth would be helpful in the future, as it would better align with their schedules and eliminate the need for travel. However, nearly half the participants agreed or strongly agreed that using telemedicine might prevent them from being properly examined, limiting their ability to communicate effectively (35).

Studies examining nurses’ perspectives on telehealth and healthcare quality have generally shown that nursing staff express positive opinions regarding telehealth interventions. They emphasized telehealth's potential to improve patients’ daily lives by offering more accessible and efficient healthcare solutions, particularly for the management of chronic illnesses. By removing geographical barriers, telehealth enables timely and personalized care, while reducing the need for frequent hospital visits. Virtual nursing clinics, as a key telehealth innovation, play a critical role in achieving these benefits, providing patients with continuous support from the comfort of their homes (36–39). However, the findings of the current study revealed a significant knowledge gap among nurses regarding virtual nursing care practices. This gap can hinder the successful implementation of telehealth services and limit their effectiveness. Nurses’ knowledge and attitudes are crucial factors that influence their willingness to adopt and utilize telehealth technologies effectively. Without sufficient understanding and proficiency, nurses may face challenges in delivering high-quality care through virtual platforms, which can negatively impact patient outcomes and satisfaction.

Although most nurses had favorable opinions of telenursing care, this study showed that their current proficiency levels needed to be improved. This is the first study on nurse professionals’ attitudes and knowledge about telenursing that was carried out in Eastern Saudi Arabia. Our study can serve as a starting point for future researchers who wish to investigate related topics. This study has certain limitations. First, the study could not demonstrate causal linkages, because it was a cross-sectional study conducted at a single institution. Second, there is limited research on the topic, as the area of virtual nursing clinics is still in its infancy. This restriction makes it difficult to gather sufficient evidence or compare study results across studies. Hence, further research on telenursing should be encouraged, and training sessions on telenursing through workshops should enhance nurses’ self-confidence in successfully conducting telenursing in cardiac care. Future research could explore how age affects the adoption of telenursing and telemedicine technologies, particularly in relation to professional experience and familiarity with technology. Finally, additional studies are required to investigate the potential influences of virtual nursing on nurses’ perceptions that go beyond their current level of understanding. Comprehending these variables can facilitate the creation of interventions or tactics aimed at enhancing comprehension while also encouraging favorable perspectives and acknowledgment of telenursing among nursing practitioners.

## 5 Conclusion & recommendation

This study examined nurses’ knowledge and attitudes toward telenursing. While most participants expressed a positive attitude towards telenursing clinics and recognized their potential to improve patient outcomes and enhance the quality of healthcare, this study also revealed that a significant proportion of nurses lacked adequate knowledge of telenursing. A smaller percentage demonstrated sufficient understanding of this area. Based on these findings, educational efforts and training programs should be implemented to enhance nurses’ understanding of telenursing and encourage their participation in the ongoing professional developments related to telenursing. Providing training to all nurses in hospital-based workshops on telenursing technologies could help reduce resistance to change, promote consistency in care delivery, and empower nurses to engage in discussions on integrating telenursing into a broader healthcare system. Additionally, the factors influencing nurses’ attitudes and knowledge toward telenursing require further investigation.

## Data Availability

The raw data supporting the conclusions of this article will be made available by the authors, without undue reservation.
